# Bloody tears and hematohidrosis in a patient of PF3 dysfunction: a case report

**DOI:** 10.4076/1757-1626-2-9029

**Published:** 2009-09-15

**Authors:** Kusum L Mishra

**Affiliations:** 1 Department of Pathology, Laboratory of Coagulation Disorder, C S M Medical University, Lucknow, Uttar Pradesh, 226 003, India

## Abstract

**Introduction:**

Abnormal bleeding may result from deficiency of one of the clotting factors.

**Case presentation:**

A 13-year-old Indian girl, presented with a history of bleeding following minor injury, epistaxis, and hematuria, bleeding from gums, subcutaneous bleeding and gastro intestinal tract bleeding. She underwent a complete coagulation profile test, CT scan and ultrasound. Platelet Factor 3 dysfunction was diagnosed to be the cause of bleeding. Patient was transfused fresh frozen plasma and platelet as part of treatment. Condition of the patient after four months of diagnosis deteriorated and she started bleeding in tears and sweat.

**Conclusion:**

A thorough examination and proper workup are necessary to determine the exact cause and rule out serious conditions.

## Introduction

Depletion of any of the clotting factor leads to bleeding from different sites. Platelet factor 3 availability (PF3-A) being an insensitive to plasma coagulation factors are highly specific and reproducible [1]. Clotting time of intact platelet rich plasma decreases due to the activation of Hageman and plasma thromboplastin antecedent (PTA) and from release of platelet factor 3 (PF3) in the presence of kaolin. Subconjuctival hemorrhage leading to bloody tears is common in acute Epstein Barr virus [2] in non-accidental trauma [3], factor XIII Val34Leu polymorphism [4] and idiopathic thrombocytopenic purpura [5]. Reports from different research on PF3 availability [6] (platelet coagulant activity) revealed variety of congenital and acquired disorder [7] but no report of bloody tears and bleeding sweat (Hematohidrosis) in patients with PF3 defect received yet. In the present case, medical history and clinical examination of the patient for any other pathology were negative. The appearance of spontaneous subconjunctival hemorrhage and hematohidrosis in a patient of PF3 release defect needs thorough investigation, as it can be an indication of some threatening disorder. PF3 test performed by mixing platelet rich control plasma with platelet poor test plasma and comparing the clotting time with the mixture of platelet rich test plasma and platelet poor control plasma in the absence of phospholipid. The difference between the two clotting time for more than 3 secs revealed defect in platelet factor 3 release.

## Case presentation

A 13-year-old school-going Indian girl child complained of bleeding from gums, hematuria, sub cutaneous bleeding, gastrointestinal bleeding which she regurgitates and collects in her oral cavity, prolonged bleeding after minor trauma and history of epistaxis. Patient later developed conjunctival hemorrhage leading to bloody tears and hematohidrosis. There was no family history of bleeding. The complete coagulation profile [8] is normal except PF 3 (Table 1). Platelet count is normal (1.95 lacs/cmm).

**Table 1:** Table shows patients complete coagulation profile

Investigations	Control (secs)	Test (secs)
Prothrombin Time (PT)	14	14
Activated Partial Thromboplastin Time (APTT)	24	24
Thrombin Time (TT)	11	11
Bleeding Time (BT)	2-7 minutes	2 mins 45 secs
Platelet Function (PF 3)	30	41

## Discussion

Subconjunctival hemorrhage and hematohidrosis observed as the secondary presenting clinical feature of PF3 dysfunction for the first time in the present study. Sodhi and Jose [5] earlier reported subconjunctival hemorrhage for the first time in a case of idiopathic thrombocytopenic purpura. The glycoprotein Ia/IIa of platelet membrane plays a major role in platelet function as a primary receptor for collagen [9]. Chief Medical Examiner of Rockland Country, New York [10] has explained that around the sweat glands, there are multiple blood vessels in a net-like form. Under the pressure of great stress, the vessels constrict. Then as the anxiety passes, "the blood vessels dilate to the point of rupture and the blood goes into the sweat glands." As the sweat glands are producing a lot of sweat, it pushes the blood to the surface - coming out as droplets of blood mixed with sweat. It is still not clear how PF3 dysfunction relates with bloody tears and hematohidrosis. Further research in this direction is required to unearth the reason for unusual bleeding.

## Conclusion

A case of PF3 dysfunction shows an unusual clinical secondary entity of hematohidrosis and bloody tears for the first time. It can be very complicating for the clinician. A thorough examination and proper workup are necessary to determine the exact cause and rule out serious conditions.

## Consent

Parents of the patient gave the written informed for publication of this case report. A copy of the written consent is available for review by the Editor-in-Chief of this journal.

## Competing interests

The author declares that there are no competing interests with any author.

## Authors' contributions

KLM was solely responsible for conducting, writing and editing this manuscript.

## References

[B1] Frederick SR Otto H Platelet Factor 3 in normal subjects and patients with renal failure The J of Clinical Investigation 1968 47 901 912 5641625 10.1172/JCI105782 PMC297238

[B2] Kanafani ZA Bashur Z Kani SS Acute epstein-barr virus infection causing bilateral conjunctival hemorrhages South Med J 2005 98 390 391 10.1097/01.SMJ.0000136268.72719.AF 15813169

[B3] Spitzer SG Luorno J Noel LP Isolated subconjunctival hemorrhages in nonaccidental trauma J AAPOS 2005 9 53 56 10.1016/j.jaapos.2004.10.003 15729281

[B4] Parmeggiani F Costagliola C Incorvaia C Prevalence of factor XIII Val34Leu polymorphism in patients affected by spontaneous subconjunctival hemorrhage Am J Ophthalmol 2004 138 481 484 10.1016/j.ajo.2004.03.017 15364237

[B5] Sodhi PK Jose R Subconjunctival hemorrhage:the first presenting clinical feature of idiopathic thrombocytopenic purpura Jpn J Ophthalmol 2003 47 316 318 10.1016/S0021-5155(03)00017-0 12782172

[B6] Hardisty RM Hutton RA The kaolin clotting time of platelet-rich plasma: A test of platelet factor-3 availability Brit J Haematol 1965 11 258 262 10.1111/j.1365-2141.1965.tb06586.x 14282063

[B7] Castaldi PA Larrieu MJ Caen J Availability of platelet factor 3 and activation of factor XII in thrombasthenia Nature 1965 207 422 424 10.1038/207422a0 5885863

[B8] Pitney WR Brozovic M Dacie JVLewis SM Practical Hematology 1984 6 Churchill Livingstone 259 266

[B9] Moshfegh K Wuillemin WA Redondo M Lammle B Beer JH Liechti-Gallati S Meyer BJ Association of two silent polymorphisms of platelet glycoprotein Ia/IIa receptor with risk of myocardial infarction: A case-control study Lancet 1999 353 351 354 10.1016/S0140-6736(98)06448-4 9950439

[B10] Frederick Z Sweating Blood Annacheriyan-ga 2006 34 14 16

